# The mediating effect of social intelligence in the association between social anxiety and mental health among Chinese nursing students

**DOI:** 10.1038/s41598-024-78637-3

**Published:** 2024-11-08

**Authors:** Qiangwei Bai, Zhenti Cui, Rui Hou, Jingjing Wang

**Affiliations:** School of Medicine, SIAS University, Zhengzhou, 451100 China

**Keywords:** Social anxiety, Social intelligence, Mental health, Chinese nursing students, Mediation analysis, Psychology, Health care

## Abstract

Social anxiety is highly prevalent among nursing students and is associated with poor mental health. However, the underlying mechanism in such an association remains unclear. This study aimed to examine the potential mediating role of social intelligence in the association between social anxiety and mental health using the Stress-Coping Model (SCM) as the theoretical framework. A cross-sectional study was conducted among 748 nursing students recruited from a Chinese University from December 2022 to March 2023. Students completed an online questionnaire to assess their social anxiety, social intelligence, and mental health. The PROCESS SPSS Macro (model 4) was used to test the mediation effect of social intelligence on the association between social anxiety and mental health. The results showed that nursing students had moderate levels of social anxiety, social intelligence, and mental health, which varied according to their profile characteristics. Social anxiety was negatively associated with mental health, and the association was partially mediated by social intelligence. Our study offers fresh insights into the impact of social anxiety on mental health and sheds light on the intricate mediating role of social intelligence. These findings offer valuable insights for research and clinical endeavors aimed at formulating psychosocial interventions to enhance the mental health of nursing students.

## Introduction

Social anxiety is a common mental disorder characterized by an intense fear during and avoidance of many social situations, with impaired social functioning as its hallmark symptom^1,2^. Worldwide, social anxiety is highly prevalent, with a reported lifetime prevalence across nations ranging from 0.2% in Nigeria to 12.1% in the USA^3^. Epidemiological studies show that social anxiety disproportionately affects the young population, occurring in more than 1 in 3 (36%) young people^4^. Additionally, social anxiety is more prevalent in females than males^3^. People with social anxiety often struggle with social situations and have difficulty initiating and maintaining human interactions, manifested as extreme and persistent fear of embarrassment and humiliation^4^.

The impact of social anxiety is widespread, and numerous studies have demonstrated a significant association between social anxiety and mental health^5–7^. According to the World Health Organization (WHO), mental health is “a state of well-being in which the individual realizes his or her own abilities, can cope with the normal stresses of life, can work productively and fruitfully, and is able to make a contribution to his or her community”^8^. Mental health problems have become a major global health concern, with an increasing trend in their diagnoses and treatment in various populations and across countries, as evidenced by several recent literature reviews ^[9 10,11^.

Nursing is a challenging and stressful profession characterized by excessive workload, leadership/management styles, professional conflict, and the emotional cost of caring^12,13^. As the world’s largest direct care group, nurses undertake the major communication responsibilities to interact with the patients and families, and they also have to deal with relationships with other healthcare providers, which all put nurses at a high risk of emotional distress^12,13^. In addition, nursing students in nursing education programs need to receive high-intensity training and advance their interpersonal communication skills to better prepare them for their future professional nursing roles, which may produce high levels of stress and anxiety^12,13^. A study showed that 27.4% of nursing students had moderate social anxiety, and 76.8% had average professional adjustment^14^. Nursing students with social anxiety are at an increased risk of developing mental health problems such as depression, anxiety, and even suicide ideation^15^.

While the significant association between social anxiety and mental health is well-established among nursing students, few studies have explored the underlying mechanism of such an association. One potential factor that may play a crucial role in linking social anxiety and mental health is social intelligence. The concept of social intelligence was first coined by Thorndike in 1920^16^, who described it as the ability to understand and manage others and react appropriately in framing adaptive social relationships. Although various definitions have been proposed regarding social intelligence, they all share two key elements: the awareness of others and the response and adaptation to others^17^. Social intelligence is a broad term that involves multiple components, including personal attitude, social performance skills, empathetic ability, emotional expressiveness, and emotional confidence^18^. Socially intelligent people are creative and friendly people who can solve problems and tackle various tasks in social life, thus developing healthy co-existence with other people^17^. Social intelligence is culture-dependent, and different aims and objectives of the behaviors may be assumed as socially intelligent by various cultures^19^.

The essential role of social intelligence in maintaining a healthy social life has received increasing research attention. A plethora of studies have consistently shown a positive association between social intelligence and a wide range of health outcomes, such as self-esteem, resilience, and general mental health in clinical and non-clinical populations^20–22^. In addition, social intelligence is also closely associated with social anxiety since both can be understood as the interpersonal signals of one person affecting the behaviors of another^23^. People with social anxiety may lack the ability to correctly understand others’ emotions in social interactions, thus reinforcing their fear of misbehaving and leading to poor social intelligence^24^. On the other hand, people with poor social intelligence are more likely to develop psychological distress due to their inappropriate interactions with other people, leading to poor mental health^20^. However, the potential role of social intelligence linking social anxiety to mental health among nursing students has not been fully understood. Knowledge of the underlying mechanism underlying the correlation between social anxiety and mental health may be helpful in developing effective and targeted intervention programs to reduce the negative impacts of social anxiety and improve nursing students’ mental health.

To better comprehend the association between social anxiety, social intelligence, and mental health, we leveraged the Stress-Coping Model (SCM) developed by Lazarus and Folkman^25,26^ as our theoretical framework and asserted that social anxiety may affect mental health indirectly through social intelligence. The SCM posits that coping, including cognitive and behavioral responses, determines individuals’ different adaptations to the same stressful situation. According to Lazarus and Folkman^25,26^, stress results from an imbalance between perceived external or internal demands and the perceived personal and social resources to deal with them, which involves two steps: primary appraisal and secondary appraisal. Primary appraisal is the initial evaluation of whether the situation is irrelevant or stressful to one’s well-being, while secondary appraisal is the assessment of whether one’s resources can be used to adapt to the situation. Using this theoretical framework, when nursing students encounter social anxiety, they may appraise it as a stressful event and may also evaluate how much social anxiety may affect their mental health (primary appraisal). Next, the nursing students appraise what types of coping resources they have and how they can use their coping resources, such as social intelligence, to reduce the negative effects of the stressor (secondary appraisal). Lastly, their social intelligence may cope with stressors and limitations, leading to improved mental health. That is, social intelligence may function as a coping resource, reducing the effects of social anxiety on mental health in nursing students. In this situation, social intelligence functions as a mediating factor.

**Fig. 1:**
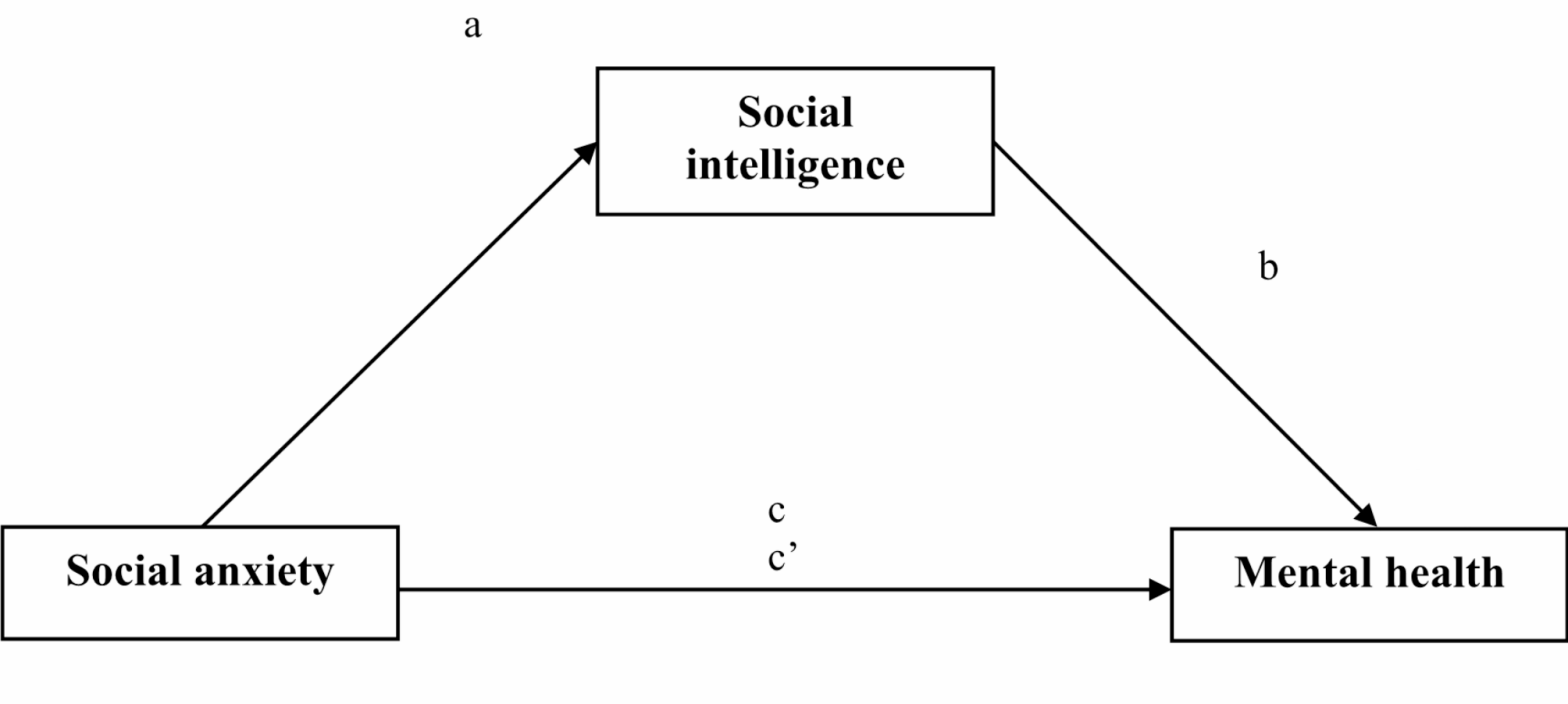
The conceptual mediation model.

Based on the SCM^25,26^, we conducted the current study to examine the mediating effect of social intelligence on the association between social anxiety and mental health among Chinese nursing students. Figure 1 shows the hypothesized conceptual mediation model of the study. Specifically, we proposed the following two hypotheses:

1. Social anxiety was negatively associated with mental health among Chinese nursing students.
2. Social intelligence mediated the association between social anxiety and mental health.

## Methods

### Study population and procedure

A cross-sectional study was conducted at a University in Henan Province, China, from December 2022 to March 2023. A convenient sampling method was utilized to recruit nursing students who satisfied the following inclusion criteria: (1) age ≥ 18, (2) nursing students enrolled in accredited nursing programs, (3) with normal cognitive and mental ability to comprehend and respond to the questionnaire, and (4) voluntary participation in the study. We excluded nursing students who had completed less than one semester of their nursing programs and those who were unable to complete the questionnaire due to severe physical and mental illness. The sample size was calculated according to the formula provided by Israel^27^: n = N * [Z2 * p * (1-p) /e2] / [N – 1 + (Z2 * p * (1-p) /e2], we set z = 2.58 (corresponding to a confidence interval of 99%), *p* = 0.5, *N* = 3,000 (population size, the total number of nursing students), and e = 0.05, which required a minimal sample size of 545. Considering a rejection rate of 25%, we further expanded our sample size to 727.

The research proposal was approved by the Ethics Review Committee of Philippine Women’s University (ERB PROTOCOL NUMBER: ERB2022_0105). The study was conducted in strict accordance with the relevant guidelines and regulations governing research involving human participants. Students were recruited by a nursing instructor in a course who provided detailed information about the study’s purpose, procedure, benefits, and potential risks. The students were informed that participation was voluntary, and refusal or dropping out of the study would not affect their study and credits in the course. The research team developed the online questionnaire using Sojump, which is China’s largest online survey platform for questionnaire design and distribution^28^. The anonymous function was activated to disable IP address tracking and ensure student privacy and anonymity. Students interested in participation could scan a QR code and access the online questionnaire after providing electronic informed consent. The online questionnaire included a request for demographic information (e.g., age and gender) and measures for social anxiety, social intelligence, and mental health, which took about 20–30 min to complete. We distributed 800 questionnaires and received 748 valid ones after eliminating invalid ones, such as those with too short completion time, with a 93.5% response rate.

### Instruments

#### Demographic information

A researcher-designed information sheet was used to collect students’ demographic information, including gender, family ranking, place of origin, personality, student leadership position, and family parenting style.

## Results

### Sample characteristics

The sample characteristics are shown in Table 1. Among the 748 students, most were females (81.8%), from rural areas (71.8%), not holding student positions (78.7%), and had introverted personalities (65.9%). Only children accounted for only 9.4% of the total sample. Regarding family parenting styles, the largest proportion of students reported democratic parenting style (46.5%), followed by dominant (29.9%) and uninvolved (17.4%) parenting styles. The mean scores of social anxiety, social intelligence, and mental health were 9.16 ± 3.83, 94.76 ± 12.99, and 16.20 ± 4.54, respectively.

Comparison of social intelligence by sample characteristics showed significantly higher scores in those with student positions (*P* < 0.001), extrovert personalities (*P* < 0.001), and democratic parenting styles (*P* = 0.016). Comparison of social anxiety by sample characteristics showed significantly higher scores in females (*P* = 0.002), those without student positions (*P* < 0.001), and those with introverted personalities (*P* < 0.001). Comparison of mental health by sample characteristics showed significantly higher scores in those with extrovert personalities (*P* < 0.001) and democratic parenting styles (*P* < 0.001).

**Table 1:** Comparison of social intelligence, social anxiety, and mental health based on sample characteristics.

Variables	Group	*N* (%)	Category (mean ± SD)								
			Social Intelligence			Social Anxiety			Mental Health		
			Score (mean ± SD)	*T/F*	*P*	Score (mean ± SD)	*T/F*	P	Score(mean ± SD)	T/F	P
Gender	Male	136 (18.3%)	96.01 ± 12.809	1.237	0.216	8.22 ± 4.069	− 3.178	0.002**	16.38 ± 5.022	0.484	0.629
	Female	612 (81.8%)	94.48 ± 13.030			9.37 ± 3.747			16.17 ± 4.432		
Place of origin	Urban	211 (28.2%)	95.99 ± 13.107	1.618	0.106	9.2 ± 4.011	0.200	0.842	16.45 ± 4.656	0.927	0.354
	Rural	537 (71.8%)	94.28 ± 12.931			9.14 ± 3.760			16.11 ± 4.497		
Holding student positions	Yes	159 (21.3%)	98.69 ± 13.240	4.350	0.000**	7.72 ± 3.768	− 5.428	0.000**	16.42 ± 4.960	0.658	0.510
	No	589 (78.7%)	93.79 ± 12.733			9.55 ± 3.757			16.15 ± 4.426		
Personality types	Extrovert	257 (34.4%)	99.86 ± 13.489	7.776	0.000**	6.98 ± 3.352	− 12.316	0.000**	17.33 ± 4.461	4.984	0.000**
	Introvert	491 (65.9%)	92.09 ± 11.901			10.30 ± 3.566			15.62 ± 4.477		
Family ranking	Has big brother/sister	247 (33.0%)	94.00 ± 11.394	0.999	0.393	9.02 ± 3.613	0.168	0.918	15.75 ± 4.545	1.855	0.136
	Has little brothers/sister	335 (44.8%)	94.64 ± 13.649			9.24 ± 3.874			16.45 ± 4.435		
	Has both big and little brother/sister	96 (12.8%)	95.70 ± 13.530			9.19 ± 3.988			16.79 ± 4.786		
	Only child	70 (9.4%)	96.74 ± 14.290			9.20 ± 4.200			15.81 ± 4.617		
	Permissive	20 (2.7%)	94.20 ± 10.675	3.054	0.016*	8.85 ± 3.703	1.609	0.170	15.20 ± 5.634	8.283	0.000**
	Authoritarian	26 (3.5%)	91.88 ± 12.401			10.15 ± 2.810			12.77 ± 4.727		
	Dominant	224 (29.9%)	93.80 ± 12.708			9.21 ± 3.460			15.72 ± 4.508		
	Uninvolved	130 (17.4%)	92.57 ± 12.985			9.69 ± 3.908			15.66 ± 4.729		
	Democratic	348 (46.5%)	96.45 ± 13.186			8.87 ± 4.077			17.03 ± 4.219		

*SD* standard deviation.

### Correlation analysis

As shown in Table 2, Pearson’s correlation analysis showed significant correlations among the participants’ social anxiety, social intelligence, and mental health. Specifically, social anxiety was negatively correlated with social intelligence (*r* = − 0.48, *P* < 0.01) and mental health (*r* = − 0.40, *P* < 0.01). In addition, social intelligence was positively correlated with mental health (*r* = 0.35, *P* < 0.01).

**Table 2:** Pearson correlation analysis.

	Social anxiety	Social intelligence	Mental health
Social anxiety	1		
Social intelligence	− 0.48**	1	
Mental health	− 0.40**	0.35**	1

**p < 0.01.

### Mediation analysis

**Fig. 2:**
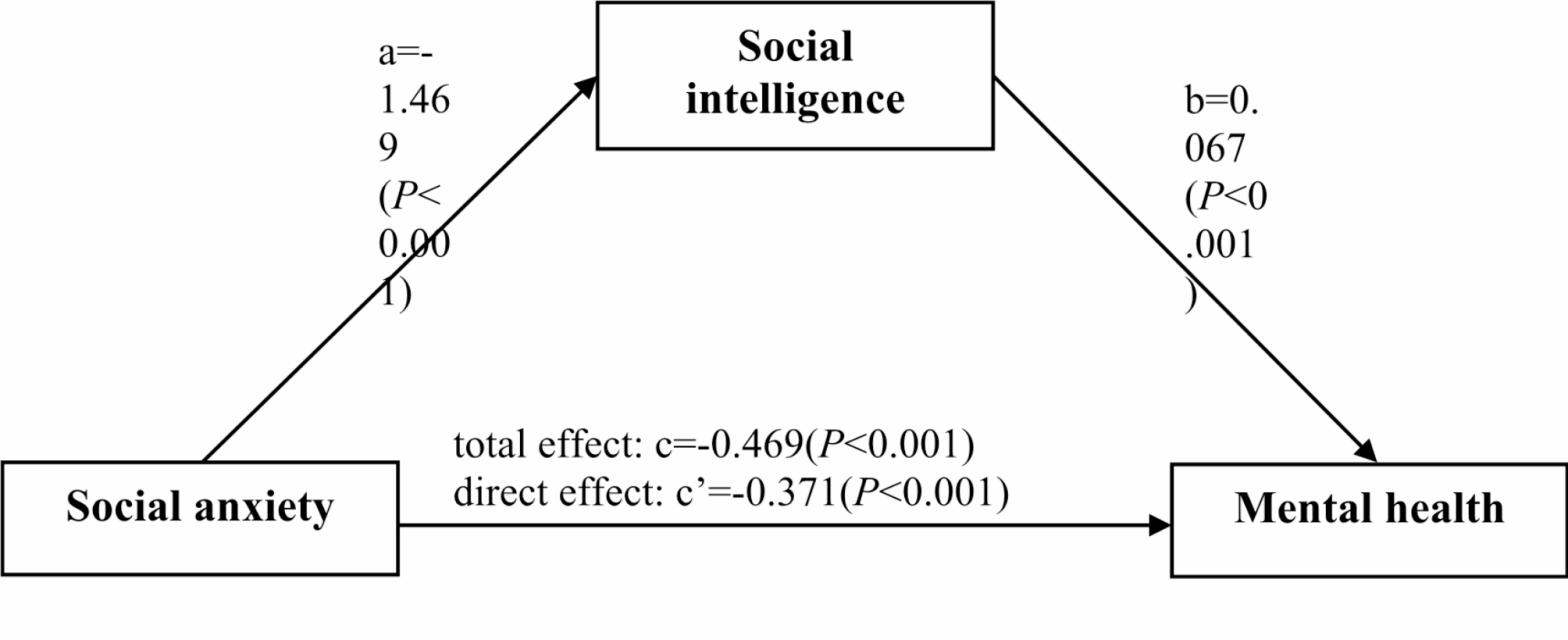
Schematic diagram of the mediation model illustrating the effect of social intelligence in the association between social anxiety and mental health. The following demographic variables were adjusted: gender, place of origin, holding student positions, personality types, family ranking.

We utilized the SPSS PROCESS v.4.3 macro (Model 4) by Hayes^36^ for mediation analysis, with social anxiety as the independent variable, mental health as the dependent variable, and social intelligence as the mediator while controlling for all sample characteristics. The results are depicted in Fig. 2; Table 3.

In assessing Hypothesis 1, a significant negative correlation was found between social anxiety and mental health (total effect c = − 0.469, 95% CI = − 0.556 to − 0.383), indicating that higher social anxiety was associated with poorer mental health. However, the correlation coefficient between social anxiety and mental health decreased from − 0.469 to − 0.371 (*p* < 0.001) after the inclusion of the mediator social intelligence, signifying partial mediation.

Upon testing Hypothesis 2, a notable indirect influence of social anxiety on mental health via social intelligence was identified (ab = − 0.098, 95% CI − 0.144 to − 0.057), indicating that higher social anxiety was associated with lower social intelligence (a = − 1.469, *p* < 0.001), which in turn, was associated with poorer mental health (b = 0.067, *p* < 0.001).

**Table 3:** Total, direct, and indirect effects of social anxiety on mental health.

	B	SE	LLCI	ULCI
Total effect (c)	− 0.469	0.044	− 0.556	− 0.383
Direct effects (c’)	− 0.371	0.047	− 0.464	− 0.278
Indirect effects (ab)	− 0.098	0.022	− 0.144	− 0.057

The number of bootstrap samples for bias-corrected bootstrap was 5,000. CI, confidence interval; LLCI, low limit confidence interval; ULCI, upper limit confidence interval.

The following demographic variables were adjusted: gender, place of origin, holding student positions, personality types, family ranking.

## Discussion

### The levels of social anxiety, social intelligence, and mental health

Our study showed that the nursing students’ social anxiety was moderate, with a mean score of 9.16 ± 3.83 out of a total score of 18. This finding was consistent with previous research indicating the high prevalence of social anxiety among Chinese university students^37,38^. The cultural emphasis on collectivism and interpersonal harmony in Chinese society, the challenges of adapting to societal changes, and the pressure to succeed may contribute to university students’ social anxiety in social interactions^39,40^. The average social intelligence score was 94.76 ± 12.99 out of a total score of 144, indicating a moderate level. Our study’s social intelligence level was higher than that reported by Zhou using the same scale (84.21 ± 3.78)^41^. This difference may be attributed to the participants’ educational background, as the current study included four-year bachelor students who may have received more humanities-related courses, potentially enhancing their social skills.

In addition, our study’s average mental health score was 16.20 ± 4.54 out of a total score of 25, indicating a moderate level. This finding aligned with previous research indicating lower mental health levels among Chinese nursing students compared to their international counterparts^42,43^. One likely explanation may be related to the COVID-19 pandemic and the stringent isolation policies in China^42^. The increasing number of confirmed COVID-19 cases and deaths, the strict prevention and control measures, and the long-term social isolation all pose potential mental health risks for college students^42^. Due to inadequate life experiences and social skills to cope with the adverse impacts of COVID-19, college students are at an increased level of psychological distress, emotional disorders, and even suicidality^44^. The adverse psychological effects related to COVID-19 and its control measures lasted even after the Chinese government started to loosen its COVID-19 control policies^44^. In addition, college students in China are under high levels of academic pressure due to the increasingly fierce competition in the job market when graduate unemployment has become a growing concern^45^. The academic pressure is even more significant in those majoring in medicine and nursing, which demand an extensive investment of time and energy to complete their study tasks, leading to even poorer mental health^46^. Furthermore, other factors such as routine-oriented activities, limited leisure time, and the impact of excessive digital device use and passive engagement with social media may also contribute to the low levels of mental health in Chinese college students^47^.

The finding that women showed higher levels of social anxiety than men aligned with previous studies highlighting gender differences in social anxiety, social skills, and emotional recognition abilities. Societal pressures may influence women’s susceptibility to social anxiety, while men’s social skills may be attributed to competitive communication strategies and assertive communication styles^48–50^. Our study showed that students holding leadership positions exhibited significantly higher levels of social intelligence and lower levels of social anxiety than students without leadership roles. These findings aligned with previous research highlighting the positive impact of leadership positions on students’ personal and social development, including the development of essential competencies such as communication, critical thinking, teamwork, and problem-solving skills^51,52^. Participation in student leadership activities has been associated with higher self-esteem, life satisfaction, lower stress levels, and better mental health^51,52^.

Furthermore, our study showed that extroverted students exhibited significantly higher levels of social intelligence and mental health and lower levels of social anxiety than introverted students. This finding was congruent with previous studies demonstrating higher social engagement, better social support, and more positive perceptions of social situations among extroverted individuals, leading to better social skills, more happiness, and higher life satisfaction^53,54^. Additionally, our study indicated that parenting style had a significant influence on social intelligence and mental health, with the highest levels of social intelligence and mental health observed in students with democratic parenting styles. These findings aligned with previous research highlighting the importance of parental warmth, support, and communication in promoting positive social outcomes, while authoritarian and permissive parenting styles may have negative effects^55,56^.

### The mediating effect of social intelligence on the association between social anxiety and mental health

Our study revealed significant relationships between social anxiety, social intelligence, and mental health. Social anxiety was negatively associated with social intelligence and mental health, while social intelligence was positively related to mental health. These findings aligned with previous research highlighting the detrimental impact of social anxiety on mental health and the positive influence of social intelligence on psychological functioning^57,58^. Prior research indicates that social anxiety hampers social interactions and decreases mental health, while social intelligence enhances social connections and contributes to positive mental health^57,58^. The study emphasizes the importance of addressing social anxiety and promoting social intelligence to improve mental health among nursing students.

Based on the SCM theory, our study examined the mediating role of social intelligence in the relationship between social anxiety and mental health among Chinese nursing students. The results indicated a significant negative indirect effect of social anxiety on mental health through social intelligence as a mediator. Specifically, higher levels of social anxiety were associated with lower levels of social intelligence, which, in turn, were associated with lower levels of mental health. Our findings added further support to the SCM theoretical framework, in which nursing students encountered social anxiety as a primary stressor and evaluated its negative impact on their mental health (primary appraisal). Then, they utilized social intelligence as a coping resource to alleviate the adverse effects of the primary stressor (secondary appraisal), which ultimately led to improved mental health.

The mediation model underscores the pivotal role of social intelligence in aiding nursing students in navigating social pressures and fostering mental well-being. Social intelligence is a multi-component global capacity encompassing intellectual, personal, communicative, and behavioral traits that are closely related to individual characteristics and interpersonal relationships^59^. Social intelligence plays a crucial role in medical fields as it shapes the level of empathic tendencies and the type of interpersonal relations with the patients^60^. For nursing students, it determines the peculiarities of their interactions and communication with patients and their effectiveness in providing appropriate care^60^. Therefore, nursing students with higher levels of social intelligence have better knowledge, skills, and competency in their future professional careers.

These theoretical insights substantiate our findings and offer implications for the cultivation and improvement of social intelligence among nursing students to improve their mental health. Our results have implications for nursing education, suggesting the integration of interventions to promote social intelligence skills among students and enhance their subjective well-being. Specifically, ongoing training curriculums and educational programs related to social intelligence promotion should be provided to all nursing students to ensure sustained personal development and career improvement. Education and training not only enhance nursing students’ knowledge and skills in social interaction and interpersonal relationships but also promote confidence and competency in their future clinical work, leading to improved mental health. In addition, it is suggested that social intelligence should be included as a critical evaluation indicator for academic performance to evaluate nursing students’ level of preparation and academic readiness to become a nurse in the future. Clinical internships can benefit from addressing social anxiety and fostering social intelligence to enhance students’ well-being in healthcare settings. Integrating social intelligence development into the curriculum and evaluation can further promote nursing students’ mental health and better prepare them for their future professional practice.

The study has several limitations that need to be acknowledged. First, the cross-sectional study design precludes the establishment of causal relationships among variables, which warrants future longitudinal study designs. Second, the nursing students were recruited from one university in Henan province, China, and may not represent nursing students from other universities in other areas. Future mufti-center studies across various parts of China are needed to test our findings in a more diverse population. Third, a convenient sampling method was used to select the study settings and participants, possibly creating a selection bias. Future studies should consider using a random sampling method to get a more representative sample. Finally, the self-reported data may result in inaccurate results due to social desirability bias. Future studies should consider combining both self-reported data and objective indicators to get a more accurate assessment and increase the reliability of research results.

## Conclusions

In summary, our study showed that undergraduate nursing students had moderate levels of social anxiety, social intelligence, and mental health, which varied according to their profile characteristics. Social anxiety was significantly associated with mental health both directly and indirectly through social intelligence. It underscores how decreased social anxiety may contribute to improved social intelligence, which may further lead to improved mental health. Our study offers fresh insights into the impact of social anxiety on mental health and sheds light on the intricate mediating role of social intelligence. These findings offer valuable insights for research and clinical endeavors aimed at formulating psychosocial interventions to enhance the mental health of nursing students.
